# Introduction to the *RSC Advances* themed collection on Nanomaterials in drug delivery

**DOI:** 10.1039/d2ra90132c

**Published:** 2023-01-11

**Authors:** Hélder A. Santos, Irina N. Savina

**Affiliations:** a Department of Biomedical Engineering, University Medical Center Groningen, University of Groningen Ant. Deusinglaan 1 Groningen 9713 AV The Netherlands h.a.santos@umcg.nl; b W. J. Kolff Institute for Biomedical Engineering and Materials Science, University Medical Center Groningen, University of Groningen Ant. Deusinglaan 1 Groningen 9713 AV The Netherlands; c Drug Research Program, Division of Pharmaceutical Chemistry and Technology, Faculty of Pharmacy, University of Helsinki Helsinki FI-00014 Finland; d School of Applied Sciences, University of Brighton Cockcroft Building, Lewes Road Brighton BN2 4GJ UK i.n.savina@brighton.ac.uk

## Abstract

Professor Hélder A. Santos and Dr Irina N. Savina introduce the *RSC Advances* themed collection on *Nanomaterials in drug delivery*.
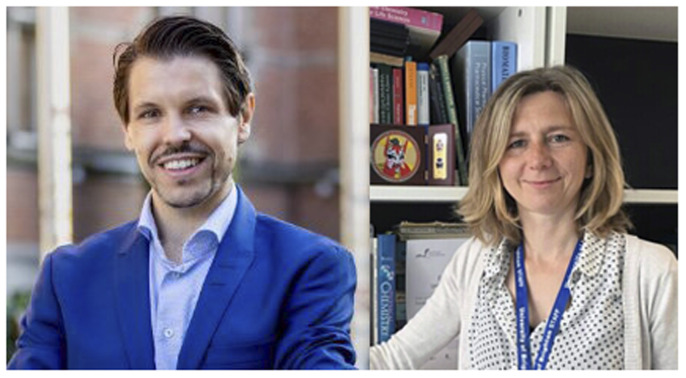

Research on nanomaterials for drug delivery applications has exponentially increased in the last few years, particularly since the impactful lipid nanocarriers used by Pfizer and Moderna were developed for severe acute respiratory syndrome coronavirus 2 (SARS-CoV-2) vaccines to treat COVID-19.^1^

There is ongoing research into the development of effective drug delivery systems that will help us deal with complex and life-threatening diseases, such as cancer, diabetes and cardiovascular diseases.^2^ Nanomaterials like liposomes, polymeric nanoparticles, metal nanoparticles, micelles, emulsions and dendrimers are becoming increasingly important in the pharmaceutical industry for improving drug formulations. The use of nanomaterials enhances the properties of conventional drugs through improved targeted drug delivery, solubility, bioavailability and drug retention time, and at the same time contributes to a reduction in side effects and risks of drug toxicity.

Nanoparticles have been produced using a variety of technologies, and particles will be formed by self-assembly, emulsification or precipitation. The choice of method is based on the ability to produce particles at the nanoscale with controlled size and good reproducibility at large scales. As an alternative to batch synthesis, microfluidic technology has been proposed, which allows better control of nanoparticle production and production on a large scale. Research continues to improve the methods available and develop more modern technologies. Grandi and co-workers demonstrated the potential of centrifugal flow-through reactors (RIACs) as a cost-effective, facile and pump-free technology for producing pharmaceutically relevant nanoparticulate systems. RIACs can be manufactured using a desktop 3D printer without post-manufacturing treatment before usage, which makes RIACs an appealing technology to research groups, especially in low-resource settings and without prior expertise in microfluidics (https://doi.org/10.1039/D2RA02745C).

Various polymers have been used for the design of nanoparticles. The main focus is on biocompatible, biodegradable, non-toxic and non-immunogenic polymers. Tortorella and co-workers review the literature work on the very recent applications of zein as an attractive and promising biopolymer for biomedical applications, and its advantageous properties in terms of shape and size, from the 1D to the final 3D perspective, including discussion of zein nanoparticles and nanocomplexes, fibers, films, membranes, microbeads, gels, and scaffolds (https://doi.org/10.1039/D1RA07424E).

Nanotechnology is key to the development of RNA therapy, which uses RNA-based delivery molecules to treat or prevent diseases that cannot be treated with conventional drugs. Recent advances in biotechnology and molecular biology make it possible to produce any peptide or protein in human cells by introducing RNA as a therapeutic agent or vaccine. The ability to produce programmed exogenous RNA and deliver it using non-viral delivery systems is more cost effective, is faster and provides flexibility in the design, something that cannot be offered by other conventional approaches. Because of that, RNA therapy can provide a quick response to the outbreak of infectious disease, such as the recent outbreak of COVID-19. RNA therapy offers hope for the development of a cure for intractable or genetic diseases. A number of RNA treatments have been successfully developed, and several clinical trials are currently underway. The review paper by Rajendran and co-workers discusses and provides an update on how mRNA therapeutics have evolved over time and the various strategies that are being explored to overcome the bottlenecks faced in utilizing mRNA as an efficient therapeutic aid, including the integration of bone tissue engineering biomaterials with mRNA for better localized delivery. The review also discusses the methods used for co-delivery of mRNA and for producing mRNA protecting proteins, and the future possibilities of utilizing mRNA therapeutics for treating various bone related genetic disorders (https://doi.org/10.1039/D2RA00713D). Research is ongoing to find more effective nanoformulations and better targeted delivery. Modification of nanoparticles with bioactive cell-recognizing molecules such as RGD improves delivery efficiency and tissue specificity in some applications (https://doi.org/10.1039/D2RA02771B). Further advances in the development of RNA drug-delivery systems will provide a solution for developing therapies for currently uncured diseases.

In the past few decades, there has been interest in using exosomes, biological nanoparticles, as novel drug delivery systems. Exosomes are cellular drug delivery systems that are used by cells to communicate and also to transport some material. As part of a cell, exosomes have low toxicity, high bioactivity, and biocompatibility. Due to their structure, exosomes do not need to be modified with specific antibodies or other biologically active molecules for targeted delivery to specific cells. Growing knowledge of the structure and biological activity of exosomes is driving researchers to develop new structures of drug delivery systems and improve existing liposome-based delivery platforms. Hybrid variants have been created in an attempt to combine the advantages of the original exosomes with the properties of synthetic systems for better and more specific drug delivery. The review paper by Lee and co-workers provided an overview of the methods for the preparation of exosome-based drug delivery systems (DDSs) through encapsulation and loading of drugs into exosomes as well as the synthesis of hybrid exosomes through diverse approaches. They also discuss the effects of treatment using exosome-based DDSs in different diseases (https://doi.org/10.1039/D2RA02351B).

This themed collection aims to explore the latest developments in the design, preparation, and application of nanomaterials for drug delivery, understand bio–nano interactions and biosystem parameters, assess the safety of nanomedicine, and assess the potential limitations of nanomedicine fabrication, including technical and legal aspects. There are currently 7 amazing contributions, including 3 review papers and 4 full papers, which broadly cover the various important topics within the field of nanomaterials in drug delivery. We would like to thank all the authors for their high-quality contributions, and we hope that researchers working in the areas of nanomaterials and drug delivery systems will enjoy reading these articles and find them useful for their future work.

## Supplementary Material
